# Pain-related support seeking? Situating the response to pain within the social context using a sample of women experiencing menstrual pain

**DOI:** 10.1007/s10865-025-00569-8

**Published:** 2025-04-26

**Authors:** Emma M. Marshall, Anat Cossen, David Skvarc, Antonina Mikocka-Walus, Marilla L. Druitt, Subhadra Evans

**Affiliations:** 1 School of Psychology, Deakin University, 75 Pigdons Road, Waurn Ponds, Geelong, VIC 3216 Australia; 2 University Hospital Geelong, Geelong, VIC Australia; 3 School of Medicine, Deakin University, Geelong, VIC Australia; 4 SEED-Lifespan, Deakin University, Geelong, VIC 3216 Australia

**Keywords:** Menstrual pain, Social support, Pain catastrophizing, Women’s health, Longitudinal

## Abstract

The current study explored whether “pain catastrophizing” in a sample of women experiencing menstrual pain can change over time as a function of perceived social support. All participants were cisgender women aged between 18 and 50 years of age, residing in Australia, and who had experienced menstruation within the past year. Participants completed two online surveys approximately 1-year apart, and participants were included if they indicated some degree of menstrual pain at baseline (Time 1, T1) (*N* = 2006 at T1 and *N* = 487 at T2). A significant negative association was found between perceived social support (T1) and pain catastrophizing (T2), when reports of pain catastrophizing at T1 were controlled for. This remained significant when the model was adjusted for the potential confounders: age, menstrual pain level, and endometriosis diagnosis (all assessed at T1). These findings demonstrate how “pain catastrophizing” in women with menstrual pain is, at least in part, a function of the broader social environment. This suggests that “pain catastrophizing” might be a well-intentioned and understandable pain-related support seeking strategy that manifests in response to a lack of adequate social support. It might be beneficial for psychological interventions to consider people’s social network to ensure that they are able to get the support that they need.

Most menstruating individuals experience menstrual pain, and while the severity of the pain varies, a concerning proportion experience moderate-to-severe levels (e.g., Parker et al., 2010; Subasinghe et al., 2016). Moderate-to-severe menstrual pain is known as dysmenorrhea, and it is estimated to affect 80% of reproductive-aged women in Australia (Subasinghe et al., 2016). This common gynecological condition occurs with or without pathological pelvic disease (e.g., endometriosis), and it has a significant impact on people’s daily functioning and costs society an estimated ~$6.5 billion per annum (Armour et al., 2019; MacGregor et al., 2023). Despite this, dysmenorrhea is poorly understood (Gagnon et al., 2022). Menstrual pain cannot be solely explained with a biomedical approach (i.e., considering biological factors alone). Instead, it is best explained with a biopsychosocial approach (i.e., considering biological, psychological, and social factors) (Chen et al., 2024; Gagnon et al., 2022). However, high quality research on the potential psychological and social mechanisms is scarce (Gagnon et al., 2022). Thus, the current study endeavors to better understand the interplay between two theoretically important psychological and social mechanisms– “pain catastrophizing” and social support (Gagon et al., 2022).

“Pain catastrophizing” has received recent attention in the menstrual pain literature (e.g., Evans et al., 2021; Gagnon et al., 2022; Rabinowitz et al., 2023). While there are various definitions of “pain catastrophizing”, it can be broadly considered to be a negatively valanced cognitive and emotional response to pain and physical symptoms (Petrini & Arendt-Nielsen, 2020 for a review). A recent meta-analysis found that “pain catastrophizing” was associated with higher ratings of menstrual pain (Rabinowitz et al., 2023) and pain relating to endometriosis more specifically (Kalfas et al., 2022). Even though the concept of “pain catastrophizing” has been around for some time, it is often poorly conceptualized and mis-understood (Petrini & Arendt-Nielsen, 2020; Sullivan & Tripp, 2024). Indeed, “pain catastrophizing” is typically labelled as a *dysfunctional* response to pain and onus for change centers around the person experiencing it (see Petrini & Arendt-Nielsen, 2020; Schutze et al., 2018; Sullivan et al., 2001). Of concern, this conceptualization might contribute to feelings of blame, dismissal, and judgment (see Connoy & Webster, 2024; Webster et al., 2023). However, it is important that researchers better situate “pain catastrophizing” within the environmental context in which it occurs.

Sullivan and colleagues’ communal coping model of pain maintains that “pain catastrophizing” is a coping strategy designed to draw others closer for the provision of care and comfort (i.e., social support) (Sullivan et al., 2001; 2012). “Pain catastrophizing” is therefore, at least in part, a well-intentioned support seeking strategy that is used in response to a social environment devoid of adequate social support (see Forest et al., 2021; Marshall et al., 2023 for aligning social support seeking literature). Following this premise, “pain catastrophizing” should manifest as a function of a person’s social environment, increasing when social support is perceived to be less than adequate. The interplay between social support and “pain catastrophizing” has received some empirical support with chronic pain patients (e.g., Carriere et al., 2020; Gauthier et al., 2011; Holtzman & DeLongis, 2007; Matthias et al., 2022; Martire et al., 2019), including people with endometriosis (Stragapede et al., 2024). Of relevance, Martire and colleagues (2019) examined a sample of osteoarthritis patients and their spouses using ecological momentary assessment and found an association between a spouses’ response to pain (punishing– a negative type of social support) and “pain catastrophizing” the next day. Interestingly, the reverse association was not found to be significant. The literature on genito-pelvic pain has also emphasized just how important a partner’s supportive (or unsupportive) responses are in explaining women’s pain experiences (see Meana & Binik, 2022 and Rosen & Bergeron, 2019 for reviews). Future research examining social support adequacy from the wider social network (i.e., friends, family, and significant others) and the role it has on “pain catastrophizing” over time is warranted. Indeed, one qualitative study has highlighted the important role that friends play in helping or hindering people adjust to chronic pain (Bernardes et al., 2023). No studies to our knowledge have examined the direct association between social support and “pain catastrophizing” using a longitudinal design. Furthermore, research is needed in the context of menstrual pain specifically.

The current prospective study aimed to explore whether “pain catastrophizing” in sample of cisgender women experiencing menstrual pain changes over time as a function of perceived social support. We hypothesized that lower levels of perceived social support adequacy at baseline (Time 1, T1) would be associated with greater levels of “pain catastrophizing” approximately 1 year later (Time 2, T2), irrespective of initial “pain catastrophizing” levels.

## Method

### Participants

Participants were cisgender women residing in Australia, who had experienced menstruation within the past year and aged between 18 and 50 years of age. Two thousand four hundred and twenty-seven participants began the T1 survey, *n* = 338 failed to provide responses to the outcome variable. Participants were asked to rate their usual level of dysmenorrhea using the item “on a scale of 0 (no pain) to 10 (worse pain possible), what is your usual level of pain during your period (without any pain medication)?” (see Evans et al., 2022). Women reporting no pain (*n* = 80) were removed. Four hundred eighty-seven participants completed the Time 2 survey. Thus, the final sample size was 2,006 at Time 1 and 487 at Time 2. The participants’ mean age at baseline was 28.6 (*SD* = 7.81) years and their mean age of first occurrence of menstruation was 12.54 (*SD* = 1.57) years and their mean menstrual pain duration was 13.09 (*SD* = 7.48) years. On average, the participants experienced dysmenorrhea (moderate-to-high levels of pain) 6.53 (*SD* = 2.24). Almost 37% of women reported having an endometriosis diagnosis.

### Procedure

The current study uses data from a larger 3-wave longitudinal study spanning 2 years (Dowding et al., 2023; Evans et al., 2022). Ethics was obtained by Deakin University’s Human Ethics Advisory Group. Participants were recruited through university forums, social media sites, and women’s gyms during May-July 2019. Participants were invited to complete an online survey hosted on Qualtrics after providing consent. Participants who completed the baseline questionnaire (Time 1, T1) between May and June 2019 and consented to be contacted for the follow-up were sent a survey link via email in June 2020 (Time 2; T2). Participants were offered the chance to win one of five $100 vouchers at baseline and one of five $50 vouchers at follow-up. The current study focuses on the first two-time points only to avoid using social support assessments over Time 2 and Time 3. This is because it was possible that these social support assessments were influenced by the unfolding COVID-19 pandemic.

### Measures

#### “Pain catastrophizing” (T1 and T2)

“Pain catastrophizing” was measured using the Pain Catastrophizing Scale (PCS), which is a 13-item self-report scale that measures one’s tendency to ruminate, exaggerate the threat value of pain, and feel helpless in the context of pain on a 0 (*not at all*) to 4 (*all the time*) scale (Sullivan et al., 1995). This scale has been used within a number of different countries and cultures, including Australia (see Ikemoto et al., 2020; Hayashi et al., 2022 for reviews). The PCS yields a total score (0–52) with a higher score indicating higher pain catastrophizing (α_Time 1_ = 0.94; α_Time 2_ = 0.94).

#### Social support (T1)

Social support was measured using the Multidimensional Scale of Perceived Social Support (MSPSS), which is a 12-item self-report scale that measures perceived social support adequacy from family, friends, and a significant other (Zimet et al., 1988). This scale has been found to have excellent reliability within an Australian context (Santiago et al., 2021). Items are rated on a 1 (*very strongly disagree*) to 7 (*very strongly agree*) scale. The MSPSS was averaged to get an overall score ranging from low (*M* = 1–2.9), through moderate (*M* = 3–5), to high (*M* = 5.1–7) support (α = 0.92).

### Data analysis

We conducted two linear regression models where the outcome, T2 “pain catastrophizing”, was regressed onto T1 “pain catastrophizing” and T1 perceived social support (indexing T1 to T2 changes in “pain catastrophizing”; Cohen & Cohen, 1983). The first model was unadjusted, so no potential confounders were included. The second model was adjusted for age, menstrual pain level at T1, and endometriosis diagnosis. These confounders were selected because they were expected to predict both social support and “pain catastrophizing” (age: Downing et al., 2022; English et al., 2014; Ruscheweyh et al., 2011; Murray et al., 2021; menstrual pain level: Cole et al., 2021; Kalfas et al., 2022; Rabinowitz et al., 2023; Stragapede et al., 2024; Walsh et al., 2003; endometriosis diagnosis: Cole et al., 2021; Evans et al., 2020; Gagnon et al., 2022; Kalfas et al., 2022; Stragapede et al., 2024). Initial inspection of data revealed no significant deviation from assumptions (independence of observations, normality of residuals, homoscedasticity, and freedom from multicollinearity). Standardized residual analysis revealed the presence of three outliers (Z > +/- 3), though these cases were uninfluential and so were retained. Analysis of the missing data suggested a possible pattern of missingness (Littles MCAR x2 (11) = 29.42, *p* =.002), though pattern inspection indicated that this was due to a small number of missing cases (< 10). Analyses were performed with and without these cases replaced through full information maximum likelihood estimation and we observed no difference in the main findings. An alpha level of 0.05 was used for all null hypothesis tests. All analyses were conducted using SPSS IBM 29.

## Results

The descriptive statistics and the bivariate correlations between the study variables are presented in Table 1. On average, participants perceived high levels of social support. “Pain catastrophizing” levels fell within a moderate range. “Pain catastrophizing” was slightly higher in T1 compared with T2. “Pain catastrophizing” at T1 and T2 was negatively associated with perceived social support.

**Table 1:** *Descriptive statistics and bivariate correlations between the study variables*

	M/Count	SD/%	Min-Max		1	2	3	4	5
1. Pain Catastrophizing (T1)	25.51	12.5	0–52	r					
				p					
2. Pain Catastrophizing (T2)	20.61	11.61	0–52	r	0.75**				
				p	< 0.001				
3. Perceived Social Support (T1)	5.31	1.18	1–7	r	− 0.20**	− 0.23**			
				p	< 0.001	< 0.001			
4. Age	28.6	7.81	18–50	r	− 0.24**	− 0.20**	-0.08		
				p	< 0.001	< 0.001	0.076		
5. Menstrual Pain Level (T1)	6.53	2.24	1–10	r	0.49**	0.42**	-0.07	− 0.12**	
				p	< 0.001	< 0.001	0.106	0.009	
6. Endometriosis†				r	− 0.29**	− 0.24**	-0.05	− 0.17**	− 0.42**
Yes	743	37%		p	< 0.001	< 0.001	0.24	< 0.001	< 0.001
No	1263	63%							

** *p* <.001. †Correlations with Endo are Spearmans rho, others are Pearsons

The results for the unadjusted and adjusted model are reported in Table 2. In line with our hypothesis, a significant negative association was found in the unadjusted and adjusted models between T1 perceived social support and T2 “pain catastrophizing”, when reports of T1 “pain catastrophizing” were controlled for. While statistically significant, the association between perceived social support and pain catastrophizing was very small (< 1% in the adjusted model and around 4% as a bivariate correlation).

**Table 2:** *Linear unadjusted and adjusted regression models predicting reports of time 2 “pain catastrophizing”*

Unadjusted								
	B	B_LL_	B_UL_	Std. Error	β	t	p	sr2
(Constant)	32.34			2.37		13.67	< 0.001	
Perceived Social Support (T1)	-0.8	-0.21	-1.38	0.3	-0.08	-2.69	0.007	< 0.01
Pain Catastrophizing (T1)	0.72	0.66	0.77	0.03	0.73	24.14	< 0.001	0.52
	**R** ^**2**^			**Adj.R** ^**2**^	**F (df)**	**Sig**		
	0.57			0.57	319.69 (2, 484)	< 0.001		
**Adjusted**								
(Constant)	10.26			3.67		2.8	0.005	
Perceived Social Support (T1)	-0.91	-0.32	-1.49	0.3	-0.09	-3.04	0.002	< 0.01
Pain Catastrophizing (T1)	0.66	0.58	0.73	0.04	0.68	18.93	< 0.001	0.32
Menstrual Pain Level (T1)	0.33	-0.04	0.70	0.19	0.06	1.71	0.088	< 0.01
Age (T1)	-0.1	< 0.001	-0.19	0.05	-0.06	-2.03	0.043	< 0.01
Endometriosis diagnosis (T1)	-0.54	1.04	-2.12	0.81	-0.02	-0.66	0.507	< 0.01
	**R** ^**2**^			**Adj.R** ^**2**^	**F (df)**	**sig**		
	0.58			0.57	130.9 (5,481)	< 0.001		

Note. sr^2^ = semi-partial correlation. B_LL_ and B_UL_ = lower and upper limits of the 95% confidence interval for the unstandardised regression coefficient

## Discussion

The current prospective study examined a sample of cisgender women with menstrual pain to determine whether change in “pain catastrophizing” over time occurred as a function of perceived social support. Supporting our hypothesis, we found a significant association between lower levels of T1 perceived social support and greater levels of T2 “pain catastrophizing”, whilst controlling for T1 “pain catastrophizing”. Although social support was weakly associated with pain catastrophizing, the association was independent of age, menstrual pain severity, and endometriosis diagnosis. This effect was also significant despite the overall stability found in “pain catastrophizing” over time.

Of importance, the findings provide evidence that “pain catastrophizing” within a sample of women experiencing menstrual pain is, at least in part, a function of the broader social environment. It is, therefore, reasonable to suggest that “pain catastrophizing” can be a well-intentioned and/or understandable pain-related support seeking strategy in response to a lack of adequate social support. This supports prior theory (the Communal Coping Model Sullivan et al., 2000, 2001) and associated research (Matthias et al., 2022; Martire et al., 2019), which has suggested that “pain catastrophizing” is a coping strategy used to obtain social proximity and support. It also aligns with the broader evolutionary frameworks of emotion (Tooby & Cosmides, 2008) and pain more specifically (e.g., Williams, 2002), which argue that pain responses (i.e., pain related cognitions, affect, and behaviors) have some adaptive functions within a social context. Future menstrual pain research aiming to better understand “pain catastrophizing” might benefit by situating it within the broader support literature (see Collins & Feeney, 2000; Forest et al., 2021; Marshall et al., 2023). To illustrate, researchers could directly explore whether “pain catastrophizing” is a pain-specific version of hyperactivated support seeking (Marshall et al., 2023). An advantage of using the support transaction framework is that it allows researchers to take a biopsychosocial approach. To expand, researchers could consider “pain catastrophizing” in response to a lack of social support *and* other biological, psychological (stress or distress, personality and other individual differences), and social factors (e.g., relationship quality) (see Collins & Feeney, 2000 support model). Indeed, it is most likely that “pain catastrophizing” within individuals experiencing menstrual pain, occurs at the intersection of various biological, psychological, and social factors (Gagnon et al., 2022).

From a practical perspective, our findings suggest that it would be beneficial for professionals to identify and target deficits that exist within the social environment so that people get the support that they need when they experience menstrual pain. Recent dysmenorrhea research has encouraged health professionals to use psychological pain management interventions to address “pain catastrophizing” (Rabinowitz et al., 2023), however, no intervention systematically considers and/or targets the patients’ social environment to the best of our knowledge. We argue that interventions are likely to become more efficacious when they consider the broader social environment (Andermann et al., 2016). While psychological interventions show promise at reducing dysmenorrhea severity and interference (Rogers et al., 2023), we cannot be certain that these interventions effectively reduce “pain catastrophizing” more specifically (see Schutze et al., 2018). To increase support for people experiencing menstrual pain, it is important to first begin by improving menstrual pain literacy throughout the health sector and the broader community (Chen et al., 2024; Sullivan & Tripp, 2024). Indeed, and as noted by Chen et al. (2024), it is important that professionals are aware of the social factors associated with menstrual pain. This is particularly important given that menstrual pain is misunderstood and dismissed throughout the health sector and broader community (Moreno Gomez et al., 2023). It might also be beneficial for interventionists and health providers to (a) screen for a lack of social support and (b) include close family members and friends in appointments and interventions to provide menstrual education and opportunities for support exchanges and/or facilitate new supportive social connections (e.g., support group members, designated patient navigators) (Seidman et al., 2023) (also see Andermann, 2016, for a related clinical practice framework on addressing the social determinants for health). In some instances, it might also be beneficial to provide relationship education to ensure that close relationships are as supportive as possible (e.g., Rhoades, 2015).

The current study is a brief report designed to provide precedence for a new wave of biopsycho*social* research that considers “pain catastrophizing” in a sample of cisgender women experiencing menstrual pain. However, this study is not without its limitations. First, the study utilizes self-reported data, which is prone to bias. By situating “pain catastrophizing” within social support transactions, researchers can draw on relationship science methodology to design observational studies that provide more objective assessments of support seeking and support provision (e.g., Collins & Feeney, 2001). Shifting to observational methods, might also provide a more objective assessment of “pain catastrophizing”. Researchers have argued for a person-centered approach to “pain catastrophizing” that allows for expert judgement and other contextual information, which are obtainable using observational methods (see Crombez et al., 2020; Petrini & Arendt-Nielsen, 2020). Relatedly, “pain catastrophizing” slightly reduced over time (from T1 to T2). In line with this, post-hoc analyses revealed that pain levels also reduced while social support remained relatively stable. However, it is important to acknowledge that participant’s T2 pain experiences might have been influenced by COVID-19 with some studies noting benefits during this time as the demands of daily living decreased (Evans et al., 2021; Schwab et al., 2021). That being said, the current study’s aims and analyses did not allow us to speak to the cause of the “pain catastrophizing” reductions over time. Future research would benefit by directly exploring the role that the broader social environment has on “pain catastrophizing” (see Chen et al., 2024). Third, a proportion of women in this study were recruited through women’s endometriosis support groups. Although the exact number of women recruited in this way is unknown, it may have led to the selection of women who had more social support, overall. It is indeed possible that women facing more severe menstrual pain experiences are underrepresented in this study. Related to this, we recognize that menstrual pain can impact individuals with female reproductive organs, regardless of gender. Future research should include gender diverse people to understand their experiences. We also suggest caution when generalizing the study’s findings to other countries and cultures given that prior research has found cross-cultural differences in “pain catastrophizing” using the Pain Catastrophizing Scale (PCS) (e.g., Hayashi et al., 2022). Finally, while the current study focused on the role that social support had in “pain catastrophizing” etiology, future research examining the reciprocal associations is warranted (Papianou et al., 2023; Sullivan, 2012; Martire et al., 2019).

To conclude, the current study provides precedence for future research to explore “pain catastrophizing” within the context of the broader social environment. In line with prior theory (Sullivan et al., 2001; 2012), it is unlikely that “pain catastrophizing” amongst people with menstrual pain is always a dysfunction. Instead, it may be a well-intentioned way to elicit support that is lacking. Future research would benefit by applying the social support transaction literature to further understand these associations (e.g., Collins & Feeney, 2001; Forest et al., 2021; Marshall et al., 2023). It is our hope that this research will more accurately reflect the experience of menstrual pain, and remove the stigma and burden associated with “pain catastrophizing”. From a practical perspective, we encourage health professionals and the community to re-educate the experience of pain, to consider the broader social context in which it occurs. Acknowledging and targeting the social environment may offer additional benefits to interventions used in clinical practice.
